# A Novel Au@Cu_2_O-Ag Ternary Nanocomposite with Highly Efficient Catalytic Performance: Towards Rapid Reduction of Methyl Orange Under Dark Condition

**DOI:** 10.3390/nano10010048

**Published:** 2019-12-24

**Authors:** Tong Wu, Yichuan Kou, Hui Zheng, Jianing Lu, Naveen Reedy Kadasala, Shuo Yang, Chenzi Guo, Yang Liu, Ming Gao

**Affiliations:** 1 College of Physics, Jilin Normal University, Siping 136000, China; wwwtttcn@126.com (T.W.); yichuankou@163.com (Y.K.); hzzh963@126.com (H.Z.); jianing3327539943@163.com (J.L.); 2 Key Laboratory of Functional Materials Physics and Chemistry of the Ministry of Education, Jilin Normal University, Changchun 130103, China; 3 Department of Chemistry, Towson University, Towson, MD 21252, USA; naveenkadasala@gmail.com; 4 Changchun Institute of Optics, Fine Mechanics and Physics, Chinese Academy of Sciences, Changchun 130033, China; yangshuo_2011@163.com (S.Y.); guocz@ciomp.ac.cn (C.G.)

**Keywords:** Au@Cu_2_O nanocomposites, Au@Cu_2_O-Ag nanocomposites, catalytic reduction, methyl orange

## Abstract

Au@Cu_2_O core-shell nanocomposites (NCs) were synthesized by reducing copper nitrate on Au colloids with hydrazine. The thickness of the Cu_2_O shells could be varied by adjusting the molar ratios of Au: Cu. The results showed that the thickness of Cu_2_O shells played a crucial role in the catalytic activity of Au@Cu_2_O NCs under dark condition. The Au@Cu_2_O-Ag ternary NCs were further prepared by a simple galvanic replacement reaction method. Moreover, the surface features were revealed by TEM, XRD, XPS, and UV–Vis techniques. Compared with Au@Cu_2_O NCs, the ternary Au@Cu_2_O-Ag NCs had an excellent catalytic performance. The degradation of methyl orange (MO) catalyzed by Au@Cu_2_O-Ag NCs was achieved within 4 min. The mechanism study proved that the synergistic effects of Au@Cu_2_O-Ag NCs and sodium borohydride facilitated the degradation of MO. Hence, the designed Au@Cu_2_O-Ag NCs with high catalytic efficiency and good stability are expected to be the ideal environmental nanocatalysts for the degradation of dye pollutants in wastewater.

## 1. Introduction

Synthetic organic dyes and natural pigments with considerable coloring capacity are widely applied in paper, plastics, paints, cosmetics, food, textile, mining, and printing industries [1,2,3]. Azo dyes account for 60–70% of all dyes [4]. However, the direct discharge of wastewater containing azo dyes not only destroys the ecological environment but also poses a serious threat to human life and health, as reported in previous literature [4]. For example, when individuals are exposed to methyl orange (MO), a typical representative of azo dyes, it has been shown to cause physical discomfort such as increased heart rate, vomiting, and shock [5,6]. Thus, removal and degradation of azo dyes are critically important to prevent environmental pollution and protect human health [7,8].

Nowadays, a series of post/pre-treatment methods have been proposed to remove the azo dyes from the wastewater [1,9,10,11]. Porous/activated carbons have been applied as commercial adsorbents for the wastewater treatment thanks to their high capacity for adsorbing pollutants. For instance, Chen et al. prepared layered double hydroxides decorated biomass-derived porous carbons to reduce MO from aqueous solution [12]. Pargoletti et al. synthesized activated carbon-based MnO_2_ nanoparticles by hydrothermal method and realized the adsorption and degradation of MO [13]. Unfortunately, regeneration difficulties, high cost, high carbon emission, and large losses of carbon source restrict their practical application [9]. Alternatively, ZnO and TiO_2_ semiconductor photocatalysts have been extensively employed to catalyze and reduce azo dyes based on their superiority, such as nontoxicity, low cost, and availability [14]. Feng et al. synthesized Fe_3_O_4_/ZnO-graphene oxide composites by a mild hydrothermal process and achieved the photocatalytic degradation of MO [15]. Szeto et al. used TiO_2_ as photocatalysts to study the effects of different lighting and atmosphere conditions on MO destruction [16]. However, the disadvantage of semiconductor photocatalysts is that their catalytic efficiency is low [17]. Therefore, it is urgent to design a kind of efficient, environmentally friendly, and economical multifunctional nanocomposites (NCs) for removing MO from wastewater.

Recently, Au, Ag, Pt, and other noble metal nanocatalysts are recognized as promising catalysts for the removal of azo dyes by the virtue of high efficiency [18,19,20]. Among these noble metal nanomaterials, Au nanocrystals have been proven to be the optimal catalysts due to the excellent chemical and physical stability, tunable optical properties, high catalytic performances, and the surface plasmon resonance (SPR) properties [21,22,23,24]. However, to decrease surface energy, unmodified Au nanocrystals are prone to agglomerate, which impedes the catalytic active sites and leads to reduction of catalytic capacity [25]. In order to address this problem, it has been proposed to embed Au nanocrystals into specific shell matrices to form core-shell nanostructures. This not only avoids the chemical corrosion of Au nanocrystals to some extent, but also ensures that most of Au nanocrystals can take part in catalytic reaction as much as possible [26]. Noble metal-semiconductor core-shell structured NCs keep respective properties of individual components, while presenting novel properties owing to the synergistic interfacial interaction between noble metal and semiconductor [27,28,29,30]. It is unfortunate that ZnO and TiO_2_ semiconductors can only absorb and transport photons in the UV region of the solar spectrum [31]. In contrast, p-type Cu_2_O has an excellent visible light response [32,33]. A great deal of research so far has been done to improve the photocatalytic efficiencies of Au@Cu_2_O in the last few years [34]. Most studies were confined to the discussion of photocatalytic mechanism under the condition of visible light excitation. It is generally accepted that Au nanocrystals greatly promote the photocatalytic activity of Cu_2_O because Au nanocrystals can act as a charge separation booster to decrease the recombination of carriers and enhance the effective utilization of carriers under visible light irradiation [35]. Unfortunately, it is worth noting that little research work has been studied on the catalytic action of Au@Cu_2_O in complete darkness. In fact, the presence of Cu_2_O shells may also modify the catalytic features of Au cores by regulating the charge separation and transfer in the absence of light. From this point of view, the thickness of Cu_2_O shells surrounding Au cores is responsible for the catalytic abilities of Au@Cu_2_O. Apart from noble metal-semiconductor core-shell structured NCs, noble metal decorated semiconductor NCs, such as Cu_2_O-Ag, Cu_2_O@Au core/shell, Cu_2_O@Au yolk/shell, have been proposed and synthesized by the researchers to achieve the purpose of increasing the catalytic efficiency [36,37]. The generation of Schottky barrier at semiconductor and noble metal interface leads to the formation of the enriched electron regions, which improves the efficiency of electron transfer and realizes rapid degradation of the dyes [38]. More importantly, the inner semiconductor can adjust localized SPR of noble metal on the surface of semiconductor by plasmon-exciton coupling interaction, which may provide a quick route for carrier transfer and cause more active sites [39]. Hence, there is an opportunity to study the synthesis of Au@Cu_2_O-Ag NCs and their catalytic degradation of azo dyes in the absence of light.

In this paper, Au nanocrystals were prepared and then were coated with a Cu_2_O shell. The thickness of the Cu_2_O shells could be varied by changing the Au:Cu molar ratios. The influence of the thickness of Cu_2_O shells on catalytic activities and stabilities to MO with the assistance of NaBH_4_ under dark condition was investigated. Silver ions were further reduced on the Au@Cu_2_O surfaces by a galvanic replacement method and Ag nanocrystals were attached to the surfaces of Au@Cu_2_O to achieve Au@Cu_2_O-Ag NCs. Au@Cu_2_O-Ag NCs were also applied to the catalytic reduction of MO under dark condition. Furthermore, the corresponding catalytic mechanism was studied. This work introduces a new catalyst for the rapid and efficient wastewater treatment in the absence of light.

## 2. Materials and Methods

### 2.1. Materials

Copper nitrate trihydrate (Cu(NO_3_)_2_·3H_2_O), PVP, hydrazine monohydrate (N_2_H_4_·H_2_O), silver nitrate (AgNO_3_), trisodium citrate dehydrate (C_6_H_5_Na_3_O_7_·2H_2_O), methyl orange (MO), sodium borohydride (NaBH_4_), and chloroauric acid (HAuCl_4_·4H_2_O) were acquired from Sinopharm Chemical Reagent Co., Ltd. (Shanghai, China). All the chemicals were used without further purification.

### 2.2. Synthesis of Au@Cu_2_O NCs

Step I: Au colloid solution was prepared as described in the literature [40]. 100 mL of 0.01% HAuCl_4_·4H_2_O solution and 4 mL of 1% C_6_H_5_Na_3_O_7_·2H_2_O solution were heated under reflux for 10 min to achieve the Au colloid solution.

Step II: 0.5 g of PVP was dissolved in 50 mL of 0.005 M Cu(NO_3_)_2_ solution under stirring at 450 rpm. Then, a certain amount of (1, 3, 5, 7, 9, and 11 mL) Au colloid solution was added to the mixture. Subsequently, N_2_H_4_·H_2_O (34 μL, 17.5 wt%) was immediately introduced, and the mixture was stirred for 2 min. Then, the product obtained by centrifugation was washed with anhydrous ethanol and deionized water. Finally, Au@Cu_2_O NCs with different shell thickness were acquired, which were named as AC-1 NCs, AC-3 NCs, AC-5 NCs, AC-7 NCs, AC-9 NCs, and AC-11 NCs, respectively. The same procedure was used to prepare Cu_2_O nanocrystals but the Au colloid solution was not added.

### 2.3. Synthesis of Au@Cu_2_O-Ag NCs

The AC-1 NCs with the thickest Cu_2_O shell were chosen to deposit Ag nanocrystals on their surfaces. The AC-1 NCs were dispersed in 35 mL of deionized water. 200 μL of 0.004 M AgNO_3_ solution was then added to the solution with stirring. After stirring for 10 min, the prepared Au@Cu_2_O-Ag NCs were washed several times with anhydrous ethanol and deionized water. Au@Cu_2_O-Ag NCs were named as AC-1-Ag NCs.

### 2.4. Catalytic Activity Measurement

**Figure 1:**
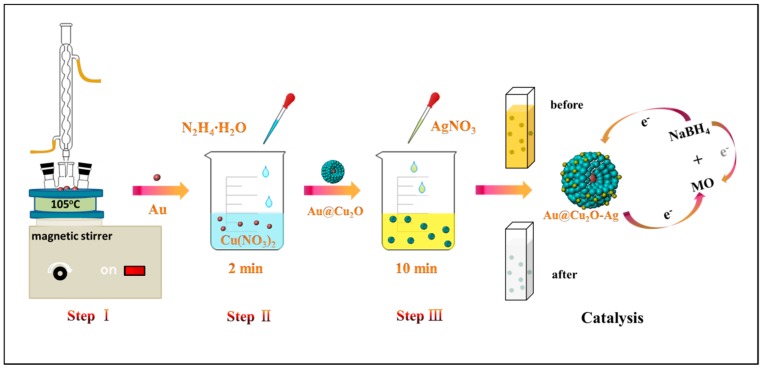
Scheme of the synthesis protocols of AC-1-Ag nanocomposites (NCs) and the degradation process for methyl orange (MO) by AC-1-Ag NCs with help of sodium borohydride (NaBH_4_).

All the catalytic activity measurements were performed in the absence of light. 2.5 mg of catalyst was added to 2.5 mL of deionized water. The catalyst was uniformly dispersed in deionized water by ultrasonication. 100 mL of 10 mg/L MO solution and 9 mL of 3.78 mg/L NaBH_4_ solution were added to the beaker. The catalyst was added for catalytic reduction and magnetic stirring was maintained during the reaction to ensure uniform dispersion. During the catalytic reduction, 2 mL of solution was sampled from the suspended catalyst nanoparticles for measurement at certain time intervals. The catalytic process was monitored by measuring the change in absorbance of MO at 464 nm by ultraviolet–visible (UV–Vis) absorption spectroscopy. To investigate the repeatability, the samples were separated by centrifugation and then washed several times. Then, the same procedure for catalytic reduction of MO solution was repeated for four cycles. Figure 1 shows the preparation process and catalytic application for degradation of MO of AC-1-Ag NCs with assistance of NaBH_4_.

### 2.5. Characterization

Transmission electron microscopy (TEM) images were obtained by using a Hitachi H-800 transmission electron microscope (JEOL Ltd., Tokyo, Japan) operating at an accelerating voltage of 200 kV. UV–Vis absorbance spectra were recorded on a Shimadzu UV 3600 spectrophotometer (Shimadzu Corporation, Tokyo, Japan) in the range of 350–800 nm. X-ray powder diffraction (XRD) analysis was conducted using a Rigaku D/MAX 3C X-ray diffractometer (Rigaku Corporation, Tokyo, Japan) with Cu Kα radiation (λ = 1.5418 Å). X-ray photoelectron spectra (XPS) analysis was performed using a Thermo Scientific ESACLAB 250 Xi A1440 system (Thermo Fisher Scientific, Waltham, MA, USA).

## 3. Results and Discussion

**Figure 2:**
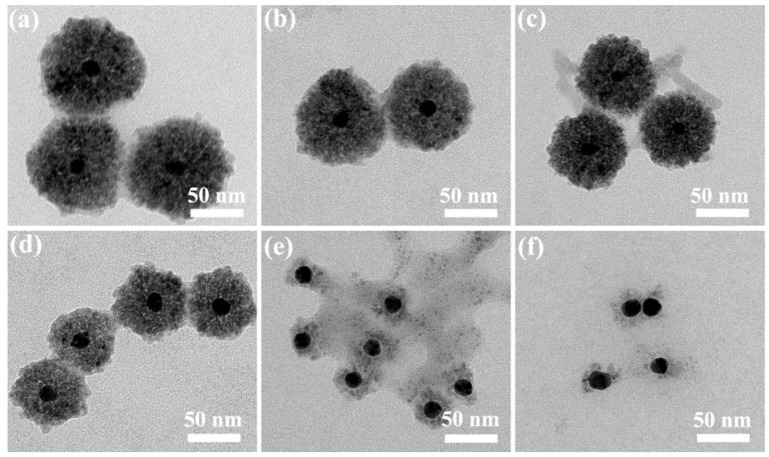
TEM images of AC-1 NCs (**a**), AC-3 NCs (**b**), AC-5 NCs (**c**), AC-7 NCs (**d**), AC-9 NCs, (**e**) and AC-11 NCs (**f**).

Figure 2 shows the TEM images of Au@Cu_2_O NCs with different Cu_2_O shell thicknesses. It is shown in Figure S1 that the average diameter of the Au core with quasi-spherical shape is about 13 nm. When the added volume of Au colloid solution is 1.00, 3.00, 5.00, and 7.00 mL, as depicted in Figure 2a–d, the Cu_2_O shell thickness of AC-1 NCs, AC-3 NCs, AC-5 NCs, and AC-7 NCs is about 82, 71, 53, and 30 nm, respectively. The surfaces of Au nanocrystals are coated by Cu_2_O to form a core-shell structure because the citrate ligands existing on the surfaces of the Au nanocrystals act as the binder and promote the contact of Cu^2+^ ions with the Au surfaces during the reduction process. The reduction of Cu^2+^ ions and the growth of Cu_2_O take place on the surfaces of Au cores [41]. As a result, Au@Cu_2_O NCs with core-shell structures are achieved. All of the four samples exhibit a relatively uniform spherical morphology and have good dispersion, which ensures that most of core-shell nanocrystals are involved in catalytic reactions. In addition, as different volumes of Au colloid solution are introduced into the Cu^2+^ reaction mixture with a fixed initial concentration, with the increase of molar ratio of Au to Cu^2+^, the Cu_2_O shell thickness attached to Au cores decreases. Similar results were found by other research groups [42,43]. Notably, when the volume of Au colloid solution is further increased to 9.00 mL, Cu_2_O shells on the surfaces of Au nanocrystals almost disappear. As for the sample of AC-11 NCs, this abnormal phenomenon is more evident. A large number of pure Au nanocrystals without Cu_2_O shells are observed, as depicted in Figure 2e,f. This is most probably because Au nanocrystals reach the supersaturation point so that there is not enough number of Cu^2+^ ions in the reaction system to form the Cu_2_O shells on the surfaces of Au nanocrystals [26].

**Figure 3:**
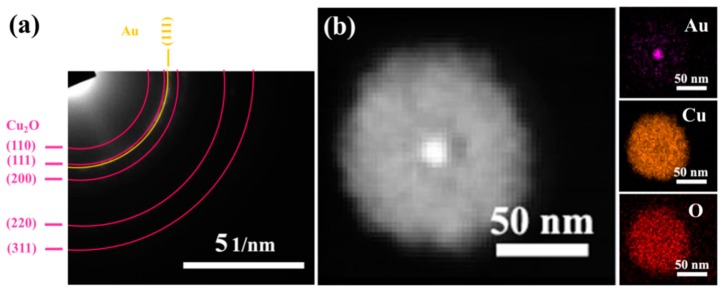
SEAD pattern (**a**) and HAADF-STEM images (**b**) of AC-1 NCs with corresponding EDS elemental mapping images (Au, Cu, and O).

A selected area electron diffraction (SAED) pattern of AC-1 NCs is shown in Figure 3a. The concentric diffraction rings are indexed to be the (110), (111), (200), (220), and (311) planes of pure Cu_2_O, and (111) plane of Au. The XRD patterns of pure Cu_2_O nanocrystals and prepared AC-1 NCs are shown in Figure S2. As for AC-1 NCs, apart from the characteristic diffraction peaks of Cu_2_O (JCPDS card no. 05-0667), the diffraction peaks appearing at 38.18, 64.57, and 77.54° are corresponding to (111), (220), and (311) of Au (JCPDS card no. 04-0784) [44,45]. Both of the SAED and XRD results confirm that the prepared AC-1 NCs consist of Cu_2_O and Au. Figure 3b shows the high-angle annular dark-field scanning TEM (HAADF-STEM) images and the corresponding energy-dispersive spectroscopy (EDS) elemental mapping images of AC-1 NCs. It is easily observed that the outer Cu_2_O completely cover the Au cores because the image contrast is brighter for the heavier element. In addition, EDS mapping images of Au, Cu, and O elements show that the Au element is located in the center of the core-shell structure, and Cu and O elements are uniformly distributed on the surfaces of Au nanocrystals [46].

**Figure 4:**
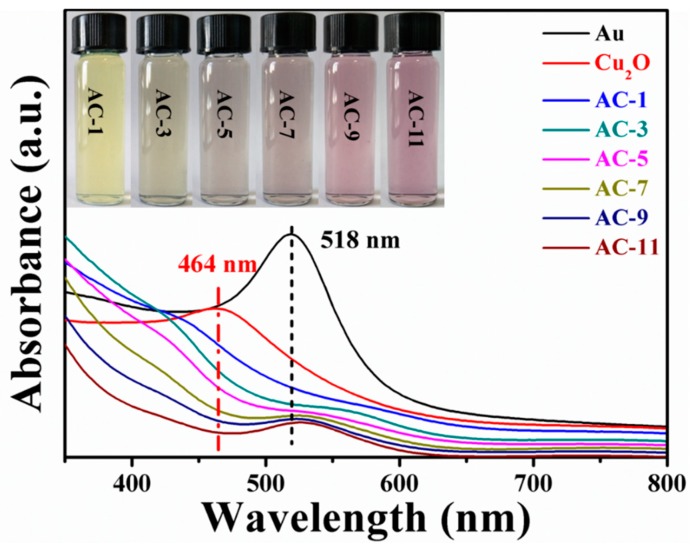
UV–Vis absorption spectra of Au colloid solution, Cu_2_O nanocrystals, AC-1 NCs, AC-3 NCs, AC-5 NCs, AC-7 NCs, AC-9 NCs, and AC-11 NCs. The inset is the photograph of AC-1 NCs, AC-3 NCs, AC-5 NCs, AC-7 NCs, AC-9 NCs, and AC-11 NCs.

Figure 4 shows the UV–Vis absorption spectra of Au colloid solution, Cu_2_O nanocrystals, and Au@Cu_2_O NCs with different shell thicknesses. The pure Au colloid solution exhibits a prominent SPR absorption peak at 518 nm. A strong absorption peak of pure Cu_2_O nanocrystals appears at 464 nm. As for Au@Cu_2_O NCs, two broad absorption peaks are observed. The absorption peak located at 526–584 nm may be attributed to plasmon resonance of Au cores, while the peak at 406–432 nm is ascribed to Cu_2_O shells. The SPR peak of Au@Cu_2_O NCs shows a significant redshift compared with that of pure Au colloid solution. With the increase of the Cu_2_O thickness, the redshift of SPR peak becomes more obvious, and the SPR peak intensity becomes weaker. A possible explanation is that Cu_2_O shells have larger refractive index compared to that of the solvent [26]. Apart from the SPR peak, it is found that there is a blueshift in the wavelength centered at 406–432 nm for Au@Cu_2_O NCs compared with the absorption peak of pure Cu_2_O nanocrystals due to the interband transition in Cu_2_O and the scattering of Cu_2_O shells [47]. It is worth emphasizing that the absorption peak of AC-9 NCs and AC-11 NCs resulting from Cu_2_O shells is almost invisible because the Cu_2_O shells are not formed on the surfaces of these two samples. The inset of Figure 4 is an optical picture of the Au@Cu_2_O NCs. The color of the solution changes from yellow to dark purple with the decrease of the Cu_2_O shell thickness. 

**Figure 5:**
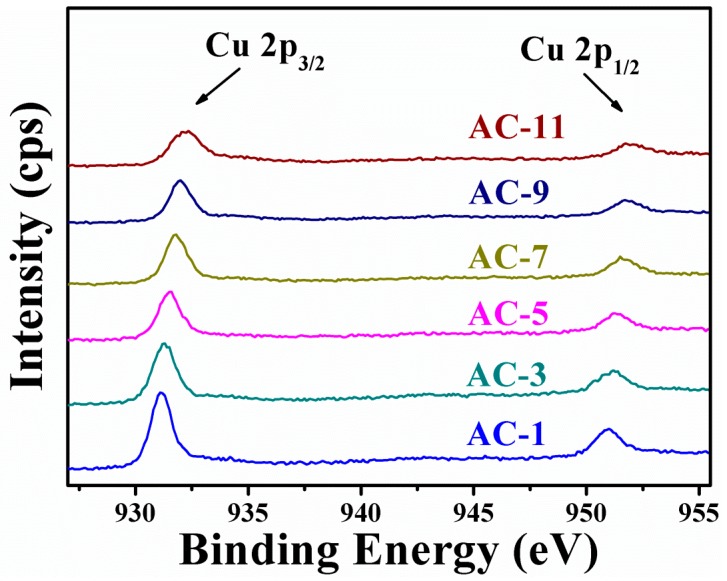
High resolution XPS scans of Cu 2p for AC-1 NCs, AC-3 NCs, AC-5 NCs, AC-7 NCs, AC-9 NCs, and AC-11 NCs.

XPS is employed to characterize the Cu oxidation state of Au@Cu_2_O NCs [48]. The high resolution XPS scans of Au@Cu_2_O NCs are presented in Figure 5. Cu 2p spectrum of AC-1 NCs exhibits two contributions assigned to Cu_2_O instead of Cu (0) or Cu (II) [49,50], which are Cu 2p_3/2_ and Cu 2p_1/2_ at 931.1 and 951.1 eV, respectively [51,52]. The peak positions of Cu 2p_3/2_ and Cu 2p_1/2_ slightly shift towards the higher binding energy side with the decrease of Cu_2_O thickness owing to the variation in the chemical environment, which demonstrates an interaction between Au and Cu_2_O [47]. In addition, because the XPS spectra intensity is directly proportional to the concentration of atoms, the binding energy intensity of Cu 2p decreases when the Cu_2_O thickness on the surfaces of Au decreases. 

**Figure 6:**
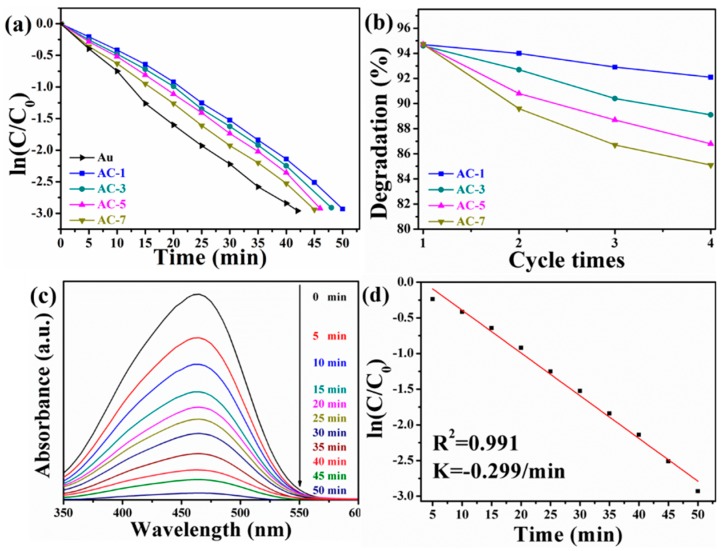
Time-dependent efficiency (**a**) of degrading MO solution catalyzed by Au nanocrystals, AC-1 NCs, AC-3 NCs, AC-5 NCs, and AC-7 NCs and recycle experiments (**b**) of degrading MO solution by AC-1 NCs, AC-3 NCs, AC-5 NCs, and AC-7 NCs. Time-dependent UV–Vis absorption spectra (**c**) and corresponding pseudo-first-order kinetic reaction plots (**d**) for reduction of MO catalyzed by AC-1 NCs.

In order to evaluate the catalytic activity of Au@Cu_2_O NCs in dark conditions, a model reaction for the degradation of MO in the presence of excess NaBH_4_ is executed by monitoring the change of the UV–Vis absorption peak at λ _max_ = 464 nm. Au nanocrystals, AC-1 NCs, AC-3 NCs, AC-5 NCs, and AC-7 NCs are used to catalyze the reduction of MO solution. Figure 6a illustrates the ln(*C*/*C*_0_) versus reaction time plots, and the degradation of MO solution is completed in 42, 50, 48, 46, and 45 min, respectively. The catalytic activity of Au nanocrystals is higher than that of Au@Cu_2_O NCs. Unfortunately, Au nanocrystals often tend to agglomerate in order to minimize their surface energy, which restricts their real application [25]. The subtle difference in reduction rate of MO by Au@Cu_2_O NCs depends upon the differences of thickness of Cu_2_O shells and size of catalysts due to the size effect [53]. Therefore, the Cu_2_O shells on the surfaces of Au nanocrystals play a crucial role in catalyzing the degradation of MO. Since the stability is an important index in appraising the quality of catalysts, the stability of Au@Cu_2_O NCs is also evaluated [54]. Four-run recycle degradation experiments in Figure 6b demonstrate that the four samples can be used for at least four successive cycles. AC-1 NCs have the highest catalytic stability. UV–Vis absorption spectra for reduction of MO catalyzed by AC-1 NCs are displayed in Figure 6c. The absorption peak at 464 nm for MO shows a downward trend and disappears within 50 min. The pseudo-first-order kinetics equation is applied to evaluate the rate constant of the reaction. The rate coefficients are obtained by using the following equation:

ln(*C/C*_0_) = −*kt*, (1)

where *C* is the concentration of MO at reaction time *t*, *C*_0_ is the initial concentration of MO at *t* = 0 and *k* is the rate constant. As shown in Figure 6d, the relationship of ln(*C/C*_0_) versus reaction time indicates that the reduction of MO catalyzed by AC-1 NCs follows the pseudo-first-order kinetics. The correlation coefficient *R^2^* and the rate constant *k* are 0.991 and 0.299 min^−1^, respectively. 

**Figure 7:**
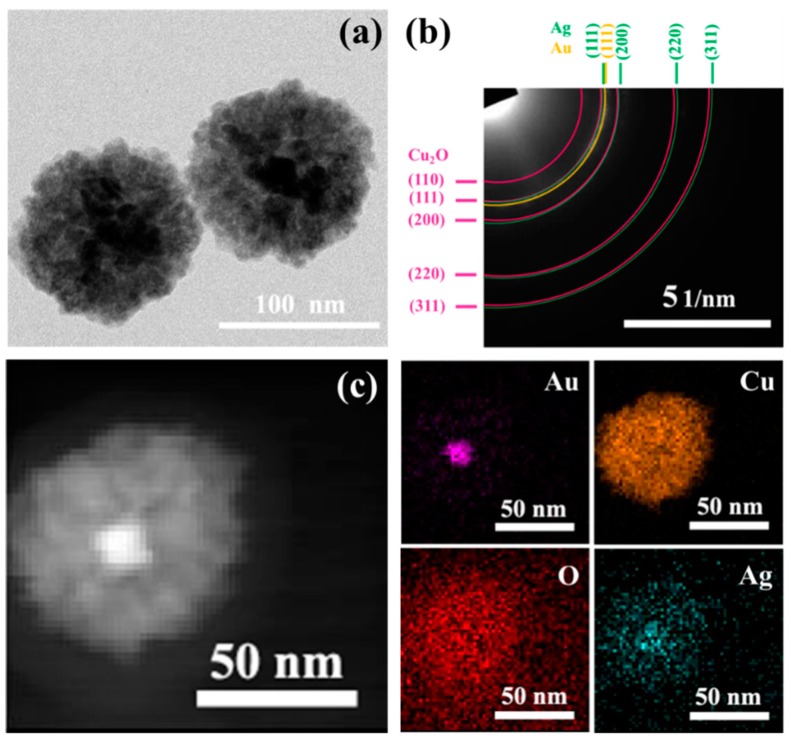
TEM image (**a**), SEAD pattern (**b**), HAADF-STEM image (**c**) of AC-1-Ag NCs with corresponding EDS elemental mapping images (Au, Cu, O, and Ag).

To enhance the catalytic performance, Ag nanocrystals are further deposited onto the surfaces of AC-1 NCs. Compared with AC-1 NCs, the TEM image of AC-1-Ag NCs in Figure 7a shows that there is no appreciable morphological change after coating with Ag nanocrystals. However, the SAED pattern of AC-1-Ag NCs in Figure 7b indicates that new diffraction rings appear, which are assigned to (111), (200), (220), and (311) crystal planes of Ag. XRD pattern of AC-1-Ag NCs in Figure S2 also confirms the existence of Ag nanocrystals. Four additional diffraction peaks located at 37.96, 44.2, 64.58, and 77.26° match the corresponding planes of Ag (JCPDS card no. 04-0783) [55,56,57]. HAADF-STEM and the corresponding EDS mapping images of AC-1-Ag NCs are presented in Figure 7c. It is observed that Ag elements are uniformly dispersed onto the surfaces of AC-1 NCs. High resolution XPS scans of Ag 3d for AC-1-Ag NCs are fitted by XPSPEAK41 software, as shown in Figure S3a. Binding energy peaks located at 368.1 and 374.1 eV with a spin-orbit splitting of 6.0 eV are ascribed to Ag 3d_5/2_ and Ag 3d_3/2_, which are consistent with the standard reference XPS spectrum of metallic Ag [58,59,60]. Figure S3b depicts UV–Vis absorption spectra of AC-1 NCs and AC-1-Ag NCs. By comparison, AC-1-Ag NCs have a strong and broad absorption peak between 310 and 480 nm, which is mainly caused by the SPR of Ag [61,62,63].

**Figure 8:**
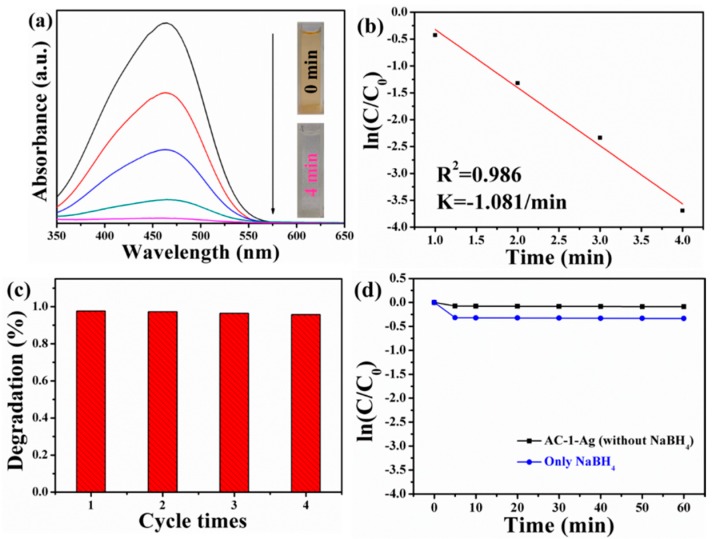
Time-dependent UV–Vis absorption spectra (**a**) and corresponding pseudo-first-order kinetic reaction plots (**b**) for reduction of MO catalyzed by AC-1-Ag NCs. Reusability of AC-1-Ag NCs for catalytic reduction of MO (**c**). Time-dependent degradation efficiency of MO solution catalyzed by AC-1-Ag NCs in different catalytic reaction conditions (**d**).

The catalytic activity of AC-1-Ag NCs in the reduction of MO is also investigated under dark condition. The UV–Vis absorption spectra and the corresponding ln(*C*/*C*_0_) versus reaction time plots of MO solution catalyzed by AC-1-Ag NCs are shown in Figure 8a,b. The complete degradation of MO solution can be realized within only 4 min. The linear relationship between logarithm of absorbance ln(*C/C*_0_) and reaction time (*t*) indicates that the reduction of MO by AC-1-Ag NCs follows pseudo-first-order kinetics. The rate constant of MO reduction is as high as 1.081 min^−1^. The above results show that the catalytic rate of AC-1-Ag NCs is significantly improved compared with that of AC-1 NCs owing to the presence of Ag nanocrystals on the surfaces of AC-1 NCs. The reusability of AC-1-Ag NCs for catalytic reduction of MO is also been evaluated, as shown in Figure 8c. The catalytic reactivity of AC-1-Ag NCs decreases slightly after four cycles, indicating that AC-1-Ag NCs are highly reusable. To better understand the possible mechanism for the reduction of MO by AC-1-Ag NCs with the help of excess NaBH_4_, MO is reduced by AC-1-Ag NCs without the addition of NaBH_4_ and by using NaBH_4_ alone. Figure 8d demonstrates negligible degradation of MO by adding only AC-1-Ag NCs and only NaBH_4_. From this point of view, both the catalysts and the electron donors (BH_4_^−^ ions) originated from NaBH_4_ are essential to reduce MO. This strongly confirms the prominent catalytic effects of AC-1-Ag NCs in the presence of an excess amount of NaBH_4_ on the reduction of MO. In fact, the kinetic barrier between acceptor molecules (MO) and BH_4_^−^ ions is too high to allow the reduction reaction of MO to proceed [64]. However, the addition of AC-1-Ag NCs provides a platform to attach BH_4_^−^ ions and MO molecules to the surfaces of AC-1-Ag NCs. In addition, the catalytic activities of AC-1-Ag NCs and Cu_2_O-Ag nanocomposites are also compared, as presented in Figure S4. The results show that AC-1-Ag NCs has better catalytic activity. The Schottky barrier existing at the interfaces of the noble metal and the semiconductor enhances the collection efficiency of carriers, but more importantly Ag nanocrystals on the surfaces and internal Au cores of AC-1-Ag NCs jointly facilitate the charge transfer from BH_4_^−^ to MO [65]. The synergistic effects of AC-1-Ag NCs and NaBH_4_ greatly promoted the catalytic efficiency of AC-1-Ag NCs. Therefore, AC-1-Ag NCs with good stability have the ability to efficiently catalyze the reduction of MO, which poses a great possibility as the hopeful nanocatalysts for application in water pollution treatment.

## 4. Conclusions

To sum up, we synthesized Au@Cu_2_O-Ag NCs by a simple galvanic replacement reaction method. The Au@Cu_2_O NCs with controllable shell thickness could be prepared by changing the amount of Au colloid solution. The results of the TEM images indicated that the shell thickness of Au@Cu_2_O NCs could be changed. It had been also demonstrated that Ag nanocrystals could be successfully compounded on Au@Cu_2_O NCs. Compared with Au@Cu_2_O NCs, the catalytic efficiency of Au@Cu_2_O-Ag NCs was significantly improved. Au@Cu_2_O-Ag NCs were capable of rapidly reducing MO within 4 min. In addition, Au@Cu_2_O-Ag NCs had good reusability and high stability. Therefore, the nanocatalysts we designed have great potential to remove organic dye pollutants from wastewater by catalytic degradation.

## Supplementary Materials

The following are available online at https://www.mdpi.com/2079-4991/10/1/48/s1. Figure S1: TEM image of the Au nanocrystals, the insert is the particle size distribution. Figure S2: XRD patterns of Cu_2_O nanocrystals, AC-1 NCs, and AC-1-Ag NCs. Figure S3: High resolution XPS scans of Ag 3d for AC-1-Ag NCs (a) and UV–Vis absorbance spectra (b) of AC-1 NCs and AC-1-Ag NCs. Figure S4: Time-dependent efficiency of degrading MO solution catalyzed by Au@Cu_2_O-Ag NCs and Cu_2_O-Ag NCs.
