# Supplementary material for: A Novel Au@Cu2O-Ag Ternary Nanocomposite with Highly Efficient Catalytic Performance: Towards Rapid Reduction of Methyl Orange Under Dark Condition
Source: Nanomaterials (Basel). 2019 Dec 24;10(1):48. doi: 10.3390/nano10010048 (PMC7023264; doi:10.3390/nano10010048)
Supplement: Supplementary file 1 [file nanomaterials-10-00048-s001.pdf]

A novel Au@Cu_2O-Ag ternary nanocomposite with highly efficient catalytic performance: Towards rapid reduction of methyl orange under dark condition

Tong Wu [1,2], Yichuan Kou [1,2], Hui Zheng [1,2], Jianing Lu [1,2], Naveen Reedy Kadasala [3], Shuo Yang [4], Chenzi Guo [4], Yang Liu [1,2,*] and Ming Gao [1,2,*]

[1] College of Physics, Jilin Normal University, Siping 136000, China; wwwtttcn@126.com (T.W.); yichuankou@163.com (Y.K.); hzzh963@126.com (H.Z.); jianing3327539943@163.com (J.L.)

[2] Key Laboratory of Functional Materials Physics and Chemistry of the Ministry of Education, Jilin Normal University, Changchun 130103, China

[3] Department of Chemistry, Towson University, Towson, MD 21252, United State; naveenkadasala@gmail.com (N.K.)

[4] Changchun Institute of Optics, Fine Mechanics and Physics, Chinese Academy of Sciences, Changchun 130033, China; yangshuo_2011@163.com (S.Y.); guocz@ciomp.ac.cn (C.G.)

* Correspondence: liuyang@jlnu.edu.cn (Y.L.); gaomingphy@126.com (M.G.); Tel./Fax: +86-434-329-4566 (Y.L. & M.G.)

Figure S1

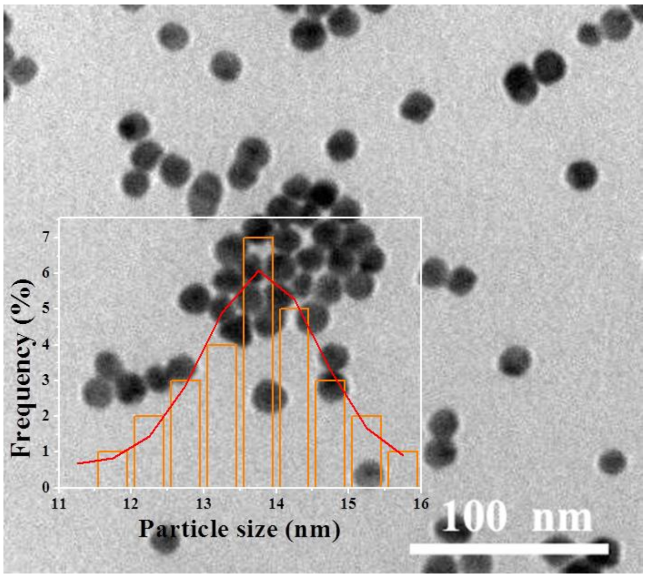

Figure S1. TEM image of the Au nanocrystals, the insert is the particle size distribution.

Figure S2

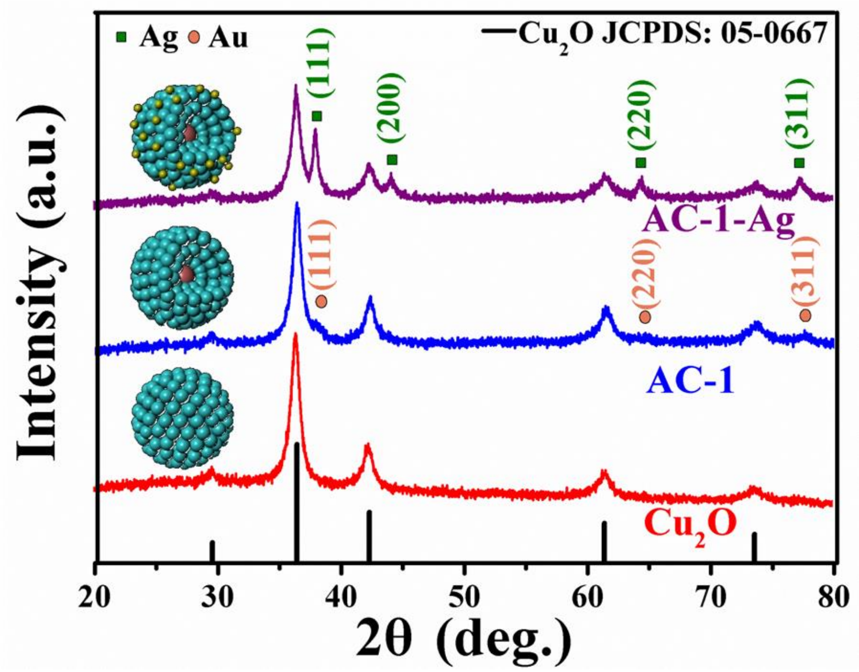

Figure S2. XRD patterns of Cu_2O nanocrystals, AC-1 NCs and AC-1-Ag NCs.

Figure S3

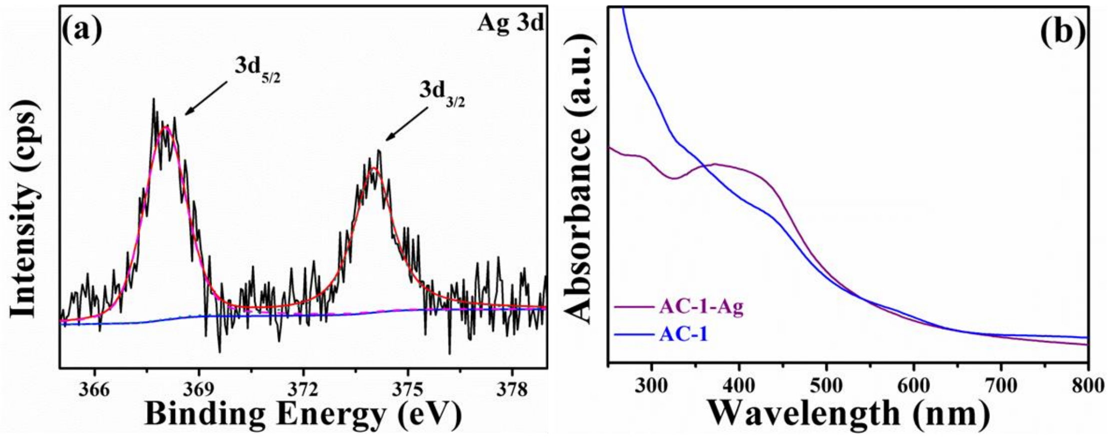

Figure S3. High resolution XPS scans of Ag 3d for AC-1-Ag NCs (a) and UV-Vis absorbance spectra (b) of AC-1 NCs and AC-1-Ag NCs.

Figure S4

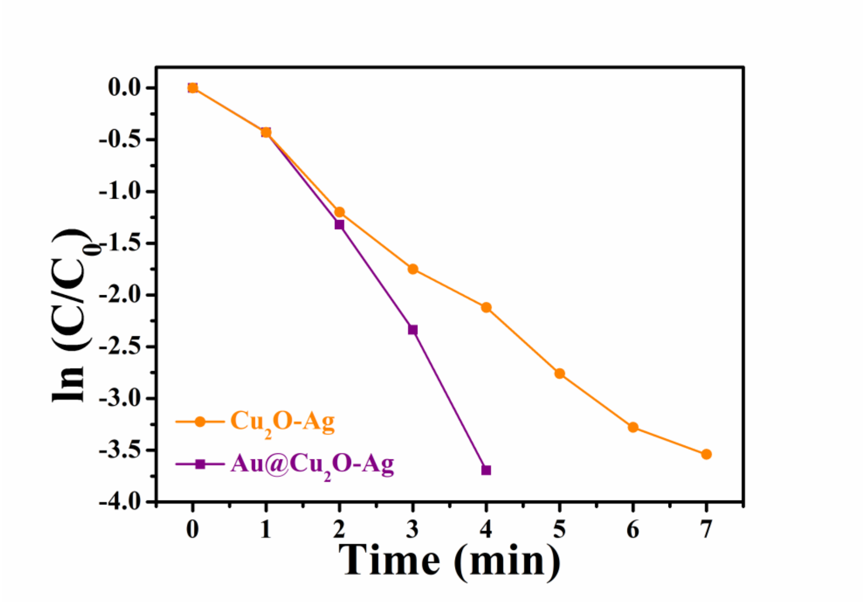

Figure S4. Time-dependent efficiency of degrading MO solution catalyzed by Au@Cu_2O-Ag NCs and Cu_2O-Ag NCs.
